# Secondary findings in 421 whole exome-sequenced Chinese children

**DOI:** 10.1186/s40246-018-0174-2

**Published:** 2018-09-14

**Authors:** Wen Chen, Wenke Li, Yi Ma, Yujing Zhang, Bianmei Han, Xuewen Liu, Kun Zhao, Meixian Zhang, Jie Mi, Yuanyuan Fu, Zhou Zhou

**Affiliations:** 1 State Key Laboratory of Cardiovascular Disease, Fuwai Hospital, National Center for Cardiovascular Diseases, Peking Union Medical College, Chinese Academy of Medical Sciences, Beijing, China; 2 State Key Laboratory of Cardiovascular Disease, Beijing Key Laboratory for Molecular Diagnostics of Cardiovascular Diseases, Diagnostic Laboratory Service, Fuwai Hospital, National Center for Cardiovascular Diseases, Peking Union Medical College, Chinese Academy of Medical Sciences, Beijing, China; 3 Department of Epidemiology, Capital Institute of Paediatrics, Beijing, China

**Keywords:** Secondary findings, Chinese children, Whole exome sequencing, Variant classification, ACMG recommendation

## Abstract

**Background:**

Variants with known or possible pathogenicity located in genes that are unrelated to primary disease conditions are defined as secondary findings. Secondary findings are not the primary targets of whole exome and genome sequencing (WES/WGS) assay but can be of great practical value in early disease prevention and intervention. The driving force for this study was to investigate the impact of racial difference and disease background on secondary findings. Here, we analyzed secondary findings frequencies in 421 whole exome-sequenced Chinese children who are phenotypically normal or bear congenital heart diseases/juvenile obesity. In total, 421 WES datasets were processed for potential deleterious variant screening. A reference gene list was defined according to the American College of Medical Genetics and Genomics (ACMG) recommendations for reporting secondary findings v2.0 (ACMG SF v2.0). The variant classification was performed according to the evidence-based guidelines recommended by the joint consensus of the ACMG and the Association for Molecular Pathology (AMP).

**Results:**

Among the 421 WES datasets, we identified 11 known/expected pathogenic variants in 12 individuals, accounting for 2.85% of our samples, which is much higher than the reported frequency in a Caucasian population. In conclusion, secondary findings are not so rare in Chinese children, which means that we should pay more attention to the clinical interpretation of sequencing results.

**Electronic supplementary material:**

The online version of this article (10.1186/s40246-018-0174-2) contains supplementary material, which is available to authorized users.

## Introduction

During whole exome and whole genome sequencing (WES/WGS) data interpretation, variants with known or possible pathogenicity located in genes that are unrelated to primary disease conditions are defined as incidental findings or secondary findings. These mutated genes are not first-tier targets of the test but indicate a high risk of certain medically actionable diseases. Hence, the early awareness of risk could be of great importance for disease prevention and intervention.

Since the American College of Medical Genetics and Genomics first published recommendations for reporting clinical exome and genome sequencing secondary findings [1], close attention has been paid to the incidence and clinical significance of secondary findings [2–7]. Considering the genetic variation of different populations and the insufficiencies in the genotype-phenotype database, technical and ethical issues regarding these findings and reports have also concerned practitioners. On the one hand, the early awareness of secondary findings provides clues for patients and clinicians to prevent diseases at a relatively early stage. On the other hand, reporting the genetic variants related to certain severe diseases before the occurrence of any phenotype may bring about an unnecessary psychological burden on individuals and even hurt the trust between patients and clinicians. The debates have never been settled, especially about the ethical issues in child patients [8].

To contribute to database accumulation and put some of those concerns to rest, we analyzed whole exome sequencing data from 421 Chinese children who were divided into phenotypically normal (96 of 421), juvenile obesity (96 of 421), and congenital heart disease (CHD) (229 of 421) groups and calculated the frequency of the secondary findings. The entire process was performed by following the ACMG recommendations for reporting secondary findings (ACMG SF v2.0) and the pathogenic classification criteria recommended by the 2015 ACMG/AMP Standards and Guidelines [9, 10]. This work is an extensive analysis of the secondary findings in Chinese children, which will provide a general view of secondary findings incidence in the Chinese population and help us to better understand the relationship between secondary findings and preexisting disease conditions. Moreover, our data analysis and interpretation could be used as a reference by other medical researchers and clinical genetic testing practitioners.

## Materials and methods

### Subject and consent

Phenotypically normal cohort and juvenile obesity cohort were recruited from the Capital Institute of Paediatrics. The CHD cohort was recruited from Fuwai Hospital. All structural heart phenotypes were confirmed by echocardiography. The WHO Child Growth Standards and International Obesity Task Force criteria were used for recruiting patients younger or older than 2 years old, respectively.

All experimental protocols were approved by the ethics committee of Fuwai Hospital and Capital Institute of Paediatrics. The guardians of the children participating in this study signed the provided written informed consent forms approved by the ethics committee of Fuwai Hospital and Capital Institute of Pediatrics and were free to quit at any stage of this study. All 421 subjects were under 18 years old at the time of sample collection. The average and median ages were 6.69 and 9 years, respectively, see Additional file 1 for the participants’ age and gender distributions.

### Whole exome sequencing

Briefly, genomic DNA was extracted from ethylenediaminetetraacetic acid (EDTA) anti-coagulated whole blood using the Wizard® Genomic DNA Purification Kit (Promega Corporation, Madison, WI, US). The quantity and quality of DNA from each sample were measured using a NanoDrop2000 (Thermo Scientific, Waltham, MA, USA). Genomic DNA was captured with NimbleGen’s SeqCap EZ Human Exome Library v3.0 kit (Roche, Pleasanton, CA, USA). Whole exome sequencing was performed on an Illumina HiSeq2500 platform (Illumina Inc., San Diego, CA, USA) using the TruSeq Rapid PE Cluster kit V2 or TruSeq Rapid SBS kit V2 - HS (Illumina Inc., San Diego, CA, USA).

### Alignment and variant calling

The Burrows-Wheeler Aligner (BWA) tool [11] was used to align the sequence reads to NCBI Build 37 (hg19). Picard tools (https://github.com/broadinstitute/picard) were used to mark the duplicate reads. The aligned sequences were then stored in BAM format and underwent variant calling using the GATK HaplotypeCaller.

### Automatic filtering

**Fig. 1:**
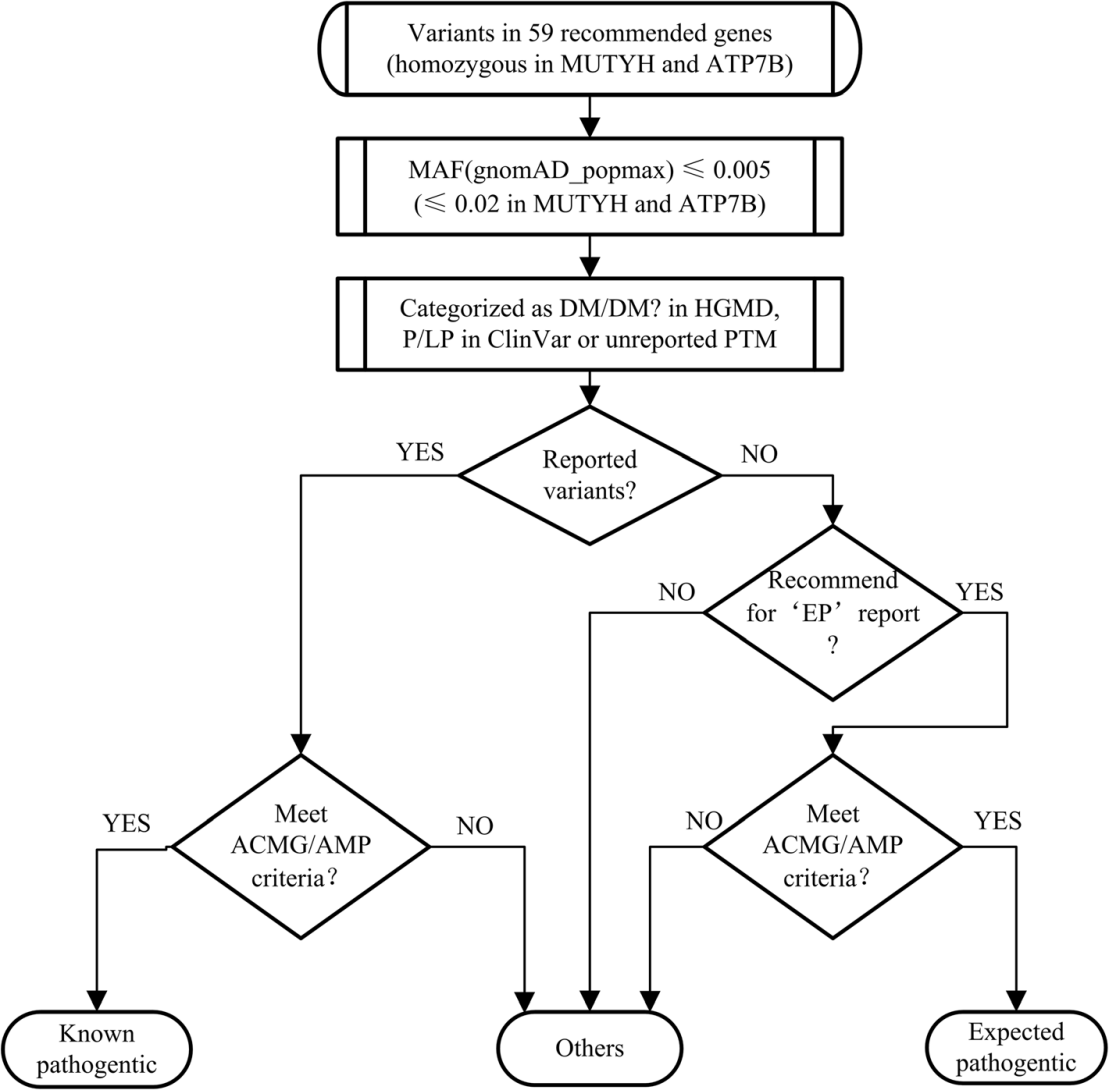
Secondary findings identification and classification workflow. Automatic filtering left 135 distinct variants for pathogenicity evaluation. According to the 2015 ACMG/AMP Standards and Guidelines, 11 variants were defined as secondary findings. SNV, single nucleotide variant; INDEL, insertion-deletion; MAF, minor allele frequencies; P/LP, pathogenic/likely pathogenic; HGMD, the Human Gene Mutation Database, professional version for release 2014.1; DM, disease-causing mutation; PTMs, protein truncating mutations; ACMG, the American College of Medical Genetics and Genomics; AMP, Association for Molecular Pathology

Variants of the 59 genes recommended by ACMG SF v2.0 were extracted. Particularly, we only kept the homozygous variants in MUTYH and ATP7B, which showed autosomal recessive inheritance. We initially extracted all rare variants defined as variants with the highest minor allele frequencies (MAFs) ≤ 0.005 in any gnomAD population (gnomAD_popmax) [12] and added the homozygote variants with the highest MAFs ≤ 0.02 in any gnomAD population for MUTYH and ATP7B. Then, the variants reported as disease-causing mutations (“DM”) or “DM?” in the Human Gene Mutation Database (HGMD), pathogenic/likely pathogenic in the ClinVar database, or protein-truncating mutations (“PTMs”), including frame-shift, stop-gain, start-loss, and splicing mutations, were kept for manual assessment. The process workflow is shown in Fig. 1.

### Manual assessment

The in silico filtered variants required evaluation for their pathogenicity according to the recommendations in the 2015 ACMG/AMP Standards and Guidelines. We also adopted the MYH7-associated inherited cardiomyopathy recommendations from ClinGen’s working group [13]. Each variant was considered to be known pathogenic (“KP”), expected pathogenic (“EP”), or “others.” The pathogenicity of each variant was evaluated according to the 2015 ACMG/AMP Standards and Guidelines. The previously reported variants that met the criteria for “pathogenic/likely pathogenic” were classified as KP. When analyzing a novel PTM, we only took the variants in genes recommended as “EP” in ACMG SF v2.0 into consideration. A category of EP would be given to a variant that met the pathogenic criteria; otherwise, it was defined as “others.” We only analyzed the pathogenicity of the reported non-PTMs; novel missense variants of these 59 genes were not considered as secondary findings according to the ACMG SF v2.0 [10], see Fig. 1 for an overview of the assessment process.

To prevent interpersonal bias, two experts specialized in data analysis and annotation simultaneously performed the assessment. When inconsistencies were present, supporting evidence was provided for further discussion and decision-making. As a final step, the lab director checked all the variant classifications along with the supporting evidence for the final reported list. All reported variants were confirmed by Sanger sequencing.

## Results

The average target region coverage was 99.54%, and 92.60% of the target regions with more than 20-fold coverage were successfully detected. The average coverage for the 59 genes was 99.74%, and 94.13% of target regions with more than 20-fold coverage were successfully detected. Specifically, the average sequencing depth of the 59 target genes was 69-fold, which contributed a reliable resource for analyzing the secondary findings. More detailed information is shown in Additional file 2.

In 421 individuals, we identified 251 rare (MAFs ≤ 0.005) variants using the 59-gene list (0.60 variants per individual). The criteria we used to classify the variants were in complete agreement with the 2015 ACMG/AMP Standards and Guidelines [9, 10]. After the automated variant filtering process, 135 unique variants remained for manual annotation, 11 of which were reportable secondary findings. Overall, we identified 5 reportable variants classified as KP or EP in the *DSP*, *MYH7*, and *FBN1* genes, which have been reported to cause different types of cardiovascular diseases. Four variants classified as KP or EP in the *BRCA2*, *PMS2*, and *SDHB* genes have been recognized to cause different types of hereditary cancer. We also identified 2 reportable variants in the *LDLR* and *APOB* genes (Table 1). Detailed evidence-based information is shown in Additional file 3. The genetic mode for all these genes is autosomal dominant inheritance with high penetrance, suggesting that the carriers are at high risk of these diseases.

**Table 1:** Variants defined as secondary findings according to the ACMG recommendations for the reportable gene list

Category	Gene	Variants	Evidence	Associated condition	Group	RCFs
Pathogenic variants:						
KP	*APOB*	NM_000384: exon26:c.10579C>T p.Arg3527Trp	PS3, PS4, PP3	Familial hypercholesterolemia	Normal/CHD	LDL-c 7.16 mmol/L 2.46 mmol/L
KP	*BRCA2*	NM_000059: exon11: c.2806-2809del p.Lys936fs	PVS1, PS4, PM2	Hereditary breast and ovarian cancer	Obesity	None
KP	*DSP*	NM_004415: exon2: c.268C>T p.Gln90Term	PVS1, PM2, PP3	Arrhythmogenic right ventricular cardiomyopathy	Obesity	None
KP	*MYH7*	NM_000257: exon18: c.1988G>A p.Arg663His	PS4, PP1_Strong, PM1, PM2, PP3	Hypertrophic cardiomyopathy, dilated cardiomyopathy	Normal	None
KP	*MYH7*	NM_000257: exon13: c.1207C>T p.Arg403Trp	PS4, PP1_Strong, PM1, PM2, PM5, PP3	Hypertrophic cardiomyopathy, dilated cardiomyopathy	Normal	None
KP	*PMS2*	NM001322010: exon8: c.498+2T>C	PVS1, PS3, PM2, PP3	Lynch syndrome	CHD	None
KP	*SDHB*	NM_003000: exon7: c.724C>T p.Arg242Cys	PS4, PM2, PM5, PP3, PS3_Supporting	Hereditary paraganglioma-pheochromocytoma syndrome	CHD	None
Likely pathogenic variants:						
KP	*LDLR*	NM_000527: exon4:c.459delC p.Phe153fs	PVS1, PM2	Familial hypercholesterolemia	Normal	LDL-c 5.13 mmol/L
KP	*MYH7*	NM_000257: exon22: c.2608C>T p.Arg870Cys	PM1, PM2, PM5, PP3, PS4_Supporting	Hypertrophic cardiomyopathy, dilated cardiomyopathy	Obesity	None
EP	*BRCA2*	NM_000059: exon11: c.2944_2945del p.Ile982fs	PVS1, PM2	Hereditary breast and ovarian cancer	Normal	None
EP	*FBN1*	NM_000138: exon25: c.3042dupT p.Ala1015fs	PVS1, PM2	Marfan syndrome, Loeys-Dietz syndromes, familial thoracic aortic aneurysms and dissections	Normal	None

*RCFs* related clinical features, *EP* expected pathogenic, *KP* known pathogenic, *ACMG* American College of Medical Genetics and Genomics, *HGMD* Human Gene Mutation Database, professional version for release 2014.1, *dbSNP* single-nucleotide polymorphism database, *CHD* congenital heart diseases, *PVS* very strong evidence of pathogenicity, *PS* strong evidence of pathogenicity, *PM* moderate evidence of pathogenicity, *PP* supporting evidence of pathogenicity, *NA* not available

According to our protocol, 135 potentially pathogenic variants were defined in 198 of 421 individuals (47.03%), and 42 of the 59 genes were affected. Among the 135 variants that required manual annotation, 34 of them were shared in two or more individuals; the remaining 101 variants were observed only once. Most of the variants were missense mutations (98/135 or 72.59%); the others included 7 synonymous variants (5.19%), 18 insertion/deletions (13.42%), 8 stop-gain/start-loss variants (5.92%), and 4 splicing region variants (2.96%). Among the affected genes, *ATP7B*, *BRCA1/2*, *MYBPC3*, and *SCN5A* bore the largest number of variations among the total variants (52/135 or 38.52%). These results may be caused by gene size and evolutionary conservation and were in accordance with Petrovski’s findings [14].

As shown in Table 1, different groups have different reportable findings, with six individuals from the phenotypically normal group, three individuals from the obesity group, and three individuals from the CHD group showing secondary findings. The reportable rates were 6.25%, 3.13%, and 1.31%, respectively. Because the patients in our cohort have not shown any of the phenotypes mentioned in the ACMG SF v2.0, all these P/LP variants were defined as secondary findings. Hence, we found 12 individuals bearing secondary findings, which accounted for 2.85% of our sample.

## Discussion

Eleven of the 135 distinct variants were reported as secondary findings after annotation and classification. Among these distinct variants, although 115 distinct variants have been reported as “potentially pathogenic” in at least one of the two most popular variation reference databases (HGMD and ClinVar), only 9 variants in 10 different individuals were finally defined as secondary findings. These results indicate that the pathogenicity of other distinct non-PTM variants in our sample might be overrated by these databases, which suggests the need to be particularly cautious when using these databases to perform clinical data interpretation. Additionally, a careful curation of these databases might be of great urgency and importance.

During the manual interpretation of these variants, in addition to the 28 criteria for classifying pathogenicity recommended by the ACMG/AMP, we also applied the latest recommendations for MYH7-associated inherited cardiomyopathies given by ClinGen’s inherited cardiomyopathy expert panel [13]. Additionally, when reporting familial hypercholesterolemia (FH)-related secondary findings, much more attention should be paid to the carriers’ conditions. Two FH-related pathogenic gene mutation carriers in our cohort had elevated LDL-c levels—7.16 and 5.13 mmol/L at the time of sample collection. The third pathogenic mutation carrier has not shown a related phenotype (LDL-c level 2.46 mmol/L). Considering the age of testing, it is understandable for such a finding.

During the past few years, many groups have focused on secondary findings reporting based on different candidate gene lists and analytical criteria. As a result, the positive rates were also different. Following the automatic filtering process and the mutation classification rules embedded in the next generation sequencing analysis workflow routines, the additional workload would be controlled at an acceptable level in actual clinical practice. Our automatic filter system called 135 distinct variants from 421 individuals, which means an average of 0.32 variants per individual during manual annotation. In comparison, the post-filtration variants per person in other studies were higher, ranging from 0.39 to 1.1 [3–5]. In our study, we tried to be more accurate and clinically practical, as shown in the following aspects: first, we adopted an authoritative and updated 59-gene list recommended by the ACMG; second, we combined the most prevalent quality control strategy, which made the remaining variants more confirmable and reliable; third, we set a stringent filter condition, which significantly reduced the workload of subsequent manual analysis; and lastly, we simplified the classification of variants into three categories (KP, EP, or “others”); thus, all we needed to consider was the ACMG pathogenicity criteria and the related published literature. The variants in the “others” category did not need to be distinguished as “variants with uncertain significance” or “(likely) benign,” which lightened the burden for laboratories.

We found a similar rate of reportable secondary findings within a family cohort [3], the Korean population [15] and East Asians [16], which is much higher than the rates in adults of European and African ancestry as reported by different groups [2, 3, 5, 17]. Several of the following reasons could explain this discrepancy: [1] the analysis was performed within different population contexts; [2] differences in the candidate gene list, variant evaluation criteria, and analytical process existed in these studies; and [3] the participants in our cohort were all relatively young (< 18 years), suggesting that some phenotypes may not show up at this age, which then led us to believe that the EP and KP variants were unrelated to primary disease and should be defined as secondary findings. Based on these assumptions, we suggest that special attention should be paid to secondary findings reporting in children and young patients.

There were also some limitations in our study. First, the samples in our cohort were from sporadic patients or phenotypically normal person and lacked family member information/samples, which made it hard for us to evaluate the pathogenicity of a de novo mutation or identify compound heterozygous/hemizygous mutations. Second, our study only included children and juvenile participants, which may have some age-related bias. Further study of random samples will be meaningful to confirm or validate our results. However, considering the unavailability of open-access Chinese population WES data, our work could reflect, to a certain degree, the secondary findings incidence in this largest population in the world.

There are always ethical issues accompanied by the interpretation of the secondary findings. Performing secondary findings on children has been a controversial topic invariably. Some groups hold the opinion that predictive testing should not be performed on children considering the consequent unrealistic burden and ethical concerns [8, 18]. However, the ACMG workgroup recommends that seeking and reporting secondary findings should not be restricted to the age of the person being sequenced [1]. The aim of secondary findings reporting is to notify the medically actionable disease-causing genetic mutation and allow the doctors and potential patients to take action in advance. From this point of view, the participants could receive additional benefit from their genomic data. To pull the two ends together, it might be more appropriate for us to respect the test receivers’ wishes, only report the portion of data they would like to know and keep the rest in the black box.

## Conclusions

In summary, by sequencing the whole exome of CHD/obesity patients and normal children, we evaluated the secondary findings incidence in Chinese minors. Secondary findings are not so rare in Chinese children, and much more attention should be paid to the clinical interpretation of genome sequencing results. Our work demonstrates some feasible interpretation criteria as well as a simplified analytical workflow for clinical practice.

## Additional files

Additional file 1: Age and gender distributions in our cohort. All 421 subjects were under 18 years old at the time of sample collection. There were more males (blue column) than females (orange column). (DOCX 20 kb)

Additional file 2: Sequencing depth and breadth of the overall target region and 59 gene-specific regions. (XLSX 61 kb)

Additional file 3: Information on known pathogenic or expected pathogenic variants according to the ACMG reportable genes. (DOCX 65 kb)

## Abbreviations

ACMG: American College of Medical Genetics and Genomics
BWA: Burrows-Wheeler Aligner
DM: Disease-causing mutation
EDTA: Ethylenediaminetetraacetic acid
EP: Expected pathogenic
FH: Familial hypercholesterolemia
KP: Known pathogenic
MAF: Minor allele frequencies
PTMs: Protein truncating mutations
WES/WGS: Whole exome and whole genome sequencing
