# Supplementary material for: Secondary findings in 421 whole exome-sequenced Chinese children
Source: Hum Genomics. 2018 Sep 14;12:42. doi: 10.1186/s40246-018-0174-2 (PMC6137878; doi:10.1186/s40246-018-0174-2)
Supplement: Supplementary file 3 — Information on known pathogenic or expected pathogenic variants according to the ACMG reportable genes. (DOCX 65 kb) [file 40246_2018_174_MOESM3_ESM.docx]

| **Gene** | **Variants** | **HGMD accession No.** | **dbSNP** | **PVS** | **PS** | **PM** | **PP** | **Category** | **References** |
| --- | --- | --- | --- | --- | --- | --- | --- | --- | --- |
| ***APOB*** | NM_000384:exon26:c.10579C>T:p.Arg3527Trp | CM950075 | rs144467873 | NA | PS3, PS4 | NA | PP3 | KP | ([1-5](#_ENREF_1)) |
| ***LDLR*** | NM_000527:exon4:c.459delC:p.Phe153fs | NA | rs1085307605 | PVS1 | NA | PM2 | NA | KP |  |
| ***MYH7*** | NM_000257:exon18:c.1988G>A:p.Arg663His | CM993620 | rs371898076 | NA | PS4, PP1_Strong | PM1, PM2 | PP3 | KP | ([6-8](#_ENREF_6)) |
| ***MYH7*** | NM_000257:exon13:c.1207C>T:p.Arg403Trp | CM930504 | rs3218714 | NA | PS4, PP1_Strong | PM1, PM2, PM5 | PP3 | KP | ([9-12](#_ENREF_9)) |
| ***BRCA2*** | BRCA2:NM_000059:exon11:c.2944_2945del:p.Ile982fs | NA | NA | PVS1 | NA | PM2 | NA | EP |  |
| ***FBN1*** | NM_000138:exon25:c.3042dupT:p.Ala1015fs | NA | NA | PVS1 | NA | PM2 | NA | EP |  |
| ***BRCA2*** | NM_000059: exon11:c.2806-2809del p.Lys936fs | NA | rs80359351 | PVS1 | PS4 | PM2 | NA | KP | ([13-15](#_ENREF_13)) |
| ***DSP*** | NM_004415:exon2:c.268C>T p.Gln90Term | NA | rs886039343 | PVS1 | NA | PM2 | PP3 | KP | ([16](#_ENREF_16), [17](#_ENREF_17)) |
| ***MYH7*** | NM_000257: exon22:c.2608C>T p.Arg870Cys | CM003687 | rs138049878 | NA | NA | PM1, PM2, PM5 | PP3, PS4_Supporting | KP | ([18](#_ENREF_18), [19](#_ENREF_19)) |
| ***PMS2*** | NM 001322010:exon8:c.498+2T>C | NA | rs878854059 | PVS1 | PS3 | PM2 | PP3 | KP | ([20-22](#_ENREF_20)) |
| ***SDHB*** | NM_003000:exon7:c.724C>T:p.Arg242Cys | CM041829 | rs786203251 | NA | PS4 | PM2, PM5 | PP3, PS3_Supporting | KP | ([23-26](#_ENREF_23)) |

Abbreviations: NA, not associated; ACMG, the American College of Medical Genetics and Genomics; EP, expected pathogenic; KP, known pathogenic; PVS, very strong evidence of pathogenicity; PS, strong evidence of pathogenicity;PM, moderate evidence of pathogenicity; PP, supporting evidence of pathogenicity;

**Reference**

1. Tai ES, Koay ES, Chan E et al. Compound heterozygous familial hypercholesterolemia and familial defective apolipoprotein B-100 produce exaggerated hypercholesterolemia. Clinical chemistry 2001: 47: 438-443.

2. Fisher E, Scharnagl H, Hoffmann MM et al. Mutations in the apolipoprotein (apo) B-100 receptor-binding region: detection of apo B-100 (Arg3500-->Trp) associated with two new haplotypes and evidence that apo B-100 (Glu3405-->Gln) diminishes receptor-mediated uptake of LDL. Clinical chemistry 1999: 45: 1026-1038.

3. Tai DY, Pan JP, Lee-Chen GJ. Identification and haplotype analysis of apolipoprotein B-100 Arg3500-->Trp mutation in hyperlipidemic Chinese. Clinical chemistry 1998: 44: 1659-1665.

4. Chiou KR, Charng MJ, Chang HM. Array-based resequencing for mutations causing familial hypercholesterolemia. Atherosclerosis 2011: 216: 383-389.

5. Hollants S, Redeker EJ, Matthijs G. Microfluidic amplification as a tool for massive parallel sequencing of the familial hypercholesterolemia genes. Clinical chemistry 2012: 58: 717-724.

6. Gruver EJ, Fatkin D, Dodds GA et al. Familial hypertrophic cardiomyopathy and atrial fibrillation caused by Arg663His beta-cardiac myosin heavy chain mutation. The American journal of cardiology 1999: 83: 13H-18H.

7. Van Driest SL, Jaeger MA, Ommen SR et al. Comprehensive analysis of the beta-myosin heavy chain gene in 389 unrelated patients with hypertrophic cardiomyopathy. Journal of the American College of Cardiology 2004: 44: 602-610.

8. Lan F, Lee AS, Liang P et al. Abnormal calcium handling properties underlie familial hypertrophic cardiomyopathy pathology in patient-specific induced pluripotent stem cells. Cell stem cell 2013: 12: 101-113.

9. Moolman JC, Brink PA, Corfield VA. Identification of a new missense mutation at Arg403, a CpG mutation hotspot, in exon 13 of the beta-myosin heavy chain gene in hypertrophic cardiomyopathy. Human molecular genetics 1993: 2: 1731-1732.

10. Dausse E, Komajda M, Fetler L et al. Familial hypertrophic cardiomyopathy. Microsatellite haplotyping and identification of a hot spot for mutations in the beta-myosin heavy chain gene. The Journal of clinical investigation 1993: 92: 2807-2813.

11. Posen BM, Moolman JC, Corfield VA et al. Clinical and prognostic evaluation of familial hypertrophic cardiomyopathy in two South African families with different cardiac beta myosin heavy chain gene mutations. British heart journal 1995: 74: 40-46.

12. Richard P, Charron P, Carrier L et al. Hypertrophic cardiomyopathy: distribution of disease genes, spectrum of mutations, and implications for a molecular diagnosis strategy. Circulation 2003: 107: 2227-2232.

13. de Juan I, Palanca S, Domenech A et al. BRCA1 and BRCA2 mutations in males with familial breast and ovarian cancer syndrome. Results of a Spanish multicenter study. Familial cancer 2015: 14: 505-513.

14. Ossa CA, Torres D. Founder and Recurrent Mutations in BRCA1 and BRCA2 Genes in Latin American Countries: State of the Art and Literature Review. The oncologist 2016: 21: 832-839.

15. Caputo S, Benboudjema L, Sinilnikova O et al. Description and analysis of genetic variants in French hereditary breast and ovarian cancer families recorded in the UMD-BRCA1/BRCA2 databases. Nucleic acids research 2012: 40: D992-1002.

16. Campuzano O, Alcalde M, Berne P et al. Role of novel DSP_p.Q986X genetic variation in arrhythmogenic right ventricular cardiomyopathy. European journal of medical genetics 2013: 56: 541-545.

17. Brun F, Barnes CV, Sinagra G et al. Titin and desmosomal genes in the natural history of arrhythmogenic right ventricular cardiomyopathy. Journal of medical genetics 2014: 51: 669-676.

18. Anan R, Shono H, Tei C. Novel cardiac beta-myosin heavy chain gene missense mutations (R869C and R870C) that cause familial hypertrophic cardiomyopathy. Human mutation 2000: 15: 584.

19. Woo A, Rakowski H, Liew JC et al. Mutations of the beta myosin heavy chain gene in hypertrophic cardiomyopathy: critical functional sites determine prognosis. Heart 2003: 89: 1179-1185.

20. Baralle D, Baralle M. Splicing in action: assessing disease causing sequence changes. Journal of medical genetics 2005: 42: 737-748.

21. Thompson BA, Spurdle AB, Plazzer JP et al. Application of a 5-tiered scheme for standardized classification of 2,360 unique mismatch repair gene variants in the InSiGHT locus-specific database. Nature genetics 2014: 46: 107-115.

22. Herkert JC, Niessen RC, Olderode-Berends MJ et al. Paediatric intestinal cancer and polyposis due to bi-allelic PMS2 mutations: case series, review and follow-up guidelines. European journal of cancer 2011: 47: 965-982.

23. Panizza E, Ercolino T, Mori L et al. Yeast model for evaluating the pathogenic significance of SDHB, SDHC and SDHD mutations in PHEO-PGL syndrome. Human molecular genetics 2013: 22: 804-815.

24. Badenhop RF, Jansen JC, Fagan PA et al. The prevalence of SDHB, SDHC, and SDHD mutations in patients with head and neck paraganglioma and association of mutations with clinical features. Journal of medical genetics 2004: 41: e99.

25. Baysal BE, Lawrence EC, Ferrell RE. Sequence variation in human succinate dehydrogenase genes: evidence for long-term balancing selection on SDHA. BMC biology 2007: 5: 12.

26. Jafri M, Whitworth J, Rattenberry E et al. Evaluation of SDHB, SDHD and VHL gene susceptibility testing in the assessment of individuals with non-syndromic phaeochromocytoma, paraganglioma and head and neck paraganglioma. Clinical endocrinology 2013: 78: 898-906.
