# Supplementary material for: Secondary findings in 421 whole exome-sequenced Chinese children
Source: Hum Genomics. 2018 Sep 14;12:42. doi: 10.1186/s40246-018-0174-2 (PMC6137878; doi:10.1186/s40246-018-0174-2)
Supplement: Supplementary file 3 [file 40246_2018_174_MOESM1_ESM.docx]

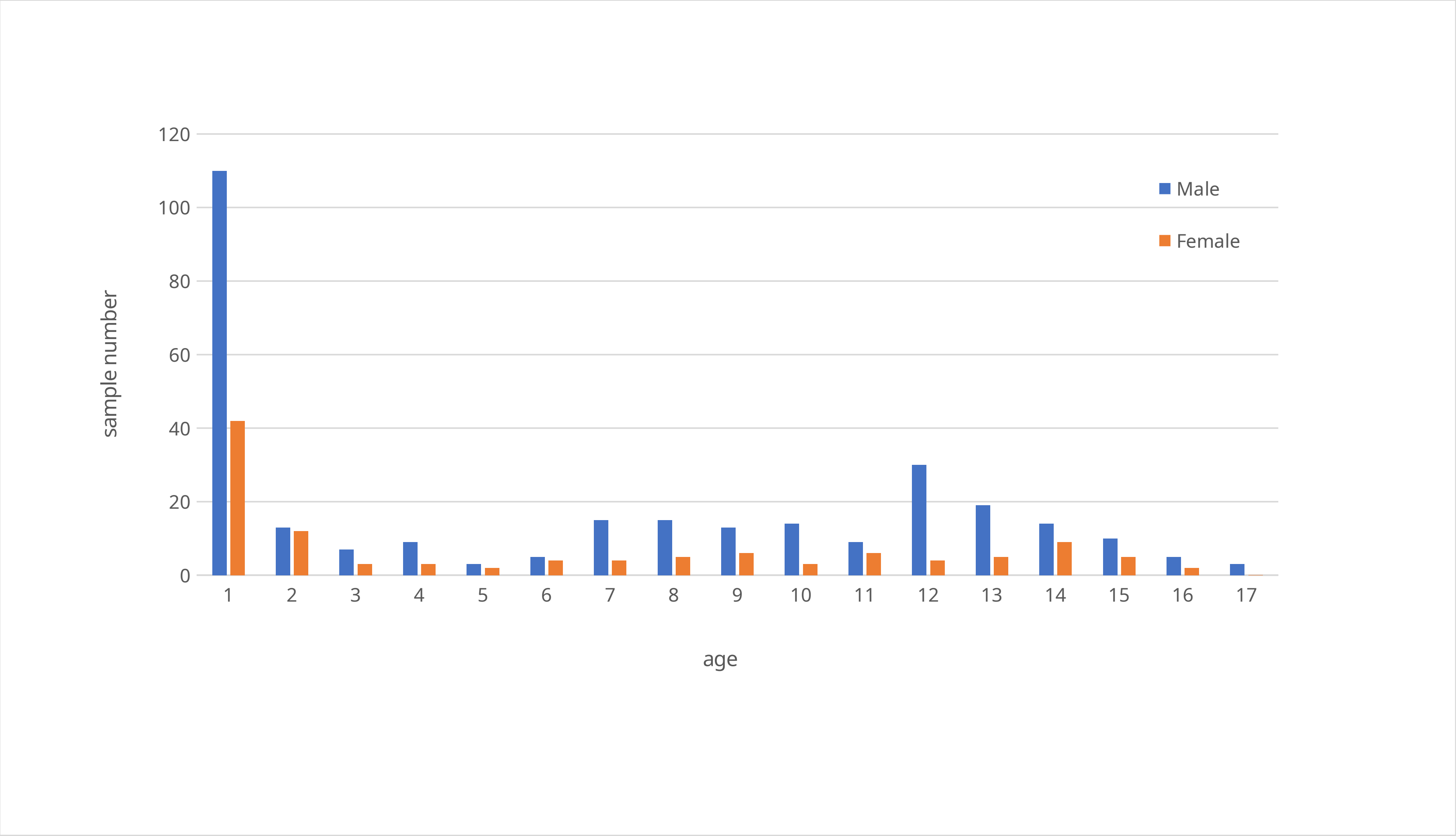
120
100
80
60
40
20
0
sample number
Male
Female
1
2
3
4
5
6
7
8
9
10
11
12
13
14
15
16
17
age
